# Isolation of α-Amylase Inhibitors from *Kadsura longipedunculata* Using a High-Speed Counter-Current Chromatography Target Guided by Centrifugal Ultrafiltration with LC-MS

**DOI:** 10.3390/molecules21091190

**Published:** 2016-09-07

**Authors:** Yin Cen, Aiping Xiao, Xiaoqing Chen, Liangliang Liu

**Affiliations:** 1 College of Chemistry and Chemical Engineering, Central South University, Changsha 410083, Hunan, China; hn_cenyin@csu.edu.cn; 2 Institute of Bast Fiber Crops, Chinese Academy of Agricultural Sciences, Changsha 410205, Hunan, China; aipingxiao@yahoo.com

**Keywords:** α-amylase, HSCCC, *Kadsuralongipedunculata*, ultrafiltration

## Abstract

In this study, a high-speed counter-current chromatography (HSCCC) separation method target guided by centrifugal ultrafiltration with high-performance liquid chromatography-mass spectrometry (CU-LC-MS) was proposed. This method was used to analyze α-amylase inhibitors from *Kadsura* *longipedunculata* extract. According to previous screening with CU-LC-MS, two screened potential α-amylase inhibitors was successfully isolated from *Kadsura* *longipedunculata* extract using HSCCC under the optimized experimental conditions. The isolated two target compounds (with purities of 92.3% and 94.6%) were, respectively, identified as quercetin-3-*O*-rhamnoside (**1**) and protocatechuic acid (**2**) based on the MS, UV, and ^1^H-NMR spectrometry data. To verify the inhibition of screened compounds, the inhibitory activities of quercetin-3-*O*-rhamnoside (**1**) and protocatechuic acid (**2**) on α-amylase were tested, and it demonstrated that the experimental IC_50_ values of quercetin-3-*O*-rhamnoside (**1**) and protocatechuic acid (**2**) were 28.8 and 12.5 μmol/L. These results proved that the hyphenated technique using CU-LC-MS and HSCCC was a rapid, competent, and reproductive method to screen and separate potential active compounds, like enzyme inhibitors from the extract of herbal medicines.

## 1. Introduction

Herbal medicines are regarded as useful resources containing variety of bioactive compounds, hence, the investigation on herbal medicines was considered as a powerful guideline for drug discovery [1,2]. However, research on herbal medicines was often hindered by arduous separation and purification procedures. Conventional unguided separation processes are time consuming, whereas the use of a bioactivity guided methods focus on only active compounds [3]. Therefore, intensive study on targeted separation combined rapid screening assays in herbal medicines was still in need [4].

Centrifugal ultrafiltration (CU) was a separation technique first proposed in the 1990s and extensively used in recent years. In this approach, compounds with high molecular weight would be retained by the membrane, and compounds with low molecular weight, including solvent, would pass through under the action of centrifugal force. The separation of compounds with different molecular weights was accomplished by adjusting the nominal molecular weight cut-off of membrane [5]. Nowadays, the combination of CU and high-performance liquid chromatography-mass spectrometry (CU-LC-MS) which allowed the affinity selection of bioactive constituents for target biomacromolecules from complexes has aroused scientific concern [6]. The screening assay via CU-LC-MS could be mainly divided into three steps, and the first step is the incubation of botanical extracts with biomacromolecules. Secondly, the CU step fosters the separation of biomacromolecules-ligand complexes from unbound small molecules and, finally, the LC-MS step analyzes and identifies the screened active compounds [7]. It has been reported that this technique has been utilized in the analysis of bioactive and active compounds, like enzyme inhibitors, protein binders, and other compounds bound to liposome and DNA [8,9,10,11,12,13], as it took advantage of low sample consumption, easy operation and simultaneous screening ability.

Developing a suitable and effective method for separation of compounds is inevitable for herbal medicine analyses. As current research progresses, conventional separation techniques, like column chromatography and thin layer chromatography, are facing similar issues, like complex processes, long processing time, and low yield. The emergence of high-speed counter-current chromatography (HSCCC) has made a breakthrough, which can eliminate the irreversible adsorption [14]. Based on the partition of compounds between two mutually immiscible liquid phases, HSCCC could effectively avert denaturation of compounds caused by solid phase. Furthermore, HSCCC could accomplish a large scale of separation with large sample loading capacity [15,16]. Nowadays, HSCCC was applied to the isolation of tea polyphones, phloroglucinolysis products, phenylethanoid glycosides, and active compounds from plants [17,18,19].

α-Amylase was widely distributed in humans and animals [20]. As a major source of the essential nutrient in foods, starch was firstly hydrolyzed by α-amylase absorption [21,22]. α-Amylase inhibitors could block the hydrolysis of carbohydrates in the human body and reduce the absorption of glucose [23]. Research on α-amylase inhibitors could promote the development in diabetes, hyperamylasemia disorders, and obesity [24]. *Kadsura* *longipedunculata* Finet et Gagnep (nanwuweizi, in Chinese) was a member of family Schisandraceae, which was widely distributed in Southern China. With such findings of active components, such as lignans and triterpenoids, from the extracts, *Kadsura longipedunculata* has demonstrated possessing the function of antimicrobial, antioxidant and the activity of antitumor and antiviral [25]. Thus, it was used in treating ulcers with pyogenic infection, rheumatoid arthritis, traumatic injury and gastrointestinal diseases [26,27]. Previous study in our lab found that the *Kadsura* *longipedunculata* extract showed inhibition on α-amylase. However, the contribution of the main components towards its inhibition activity has not been reported.

In this study, the target guided analysis method for screening and isolating α-amylase inhibitors from *Kadsura* *longipedunculata* using CU-LC-MS combined with HSCCC was established. The potential α-amylase inhibitors from *Kadsura* *longipedunculata* extract were firstly analyzed by CU-LC-MS and then target separated with HSCCC. The results yielded two active compounds, quercetin-3-*O*-rhamnoside and protocatechuic acid. The inhibitory activity of isolated compound was also estimated. It showed the isolation completed by HSCCC target guided with CU-LC-MS was convenient and efficient.

## 2. Results and Discussion

### 2.1. Screening of α-Amylase Inhibitors from Kadsura longipedunculata

**Figure 1:**
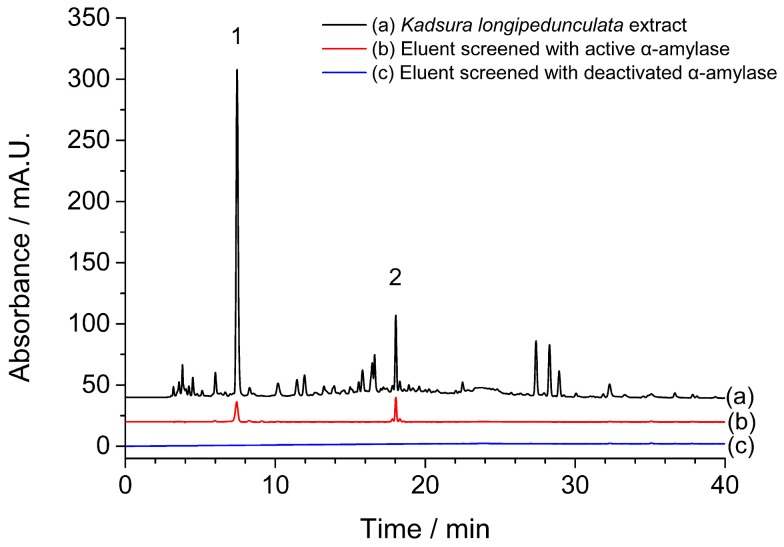
The chromatograms of (**a**) *Kadsura* *longipedunculata* before and (**b**) after performing CU with α-amylase, and (**c**) with denatured α-amylase. Two peaks appeared in the chromatogram of ultrafiltrated solvent incubated with active α-amylase were marked as 1 and 2.

Solvents with different polarities were usually used for extracting compounds from herbal medicines. Therefore, petroleum ether, ethyl acetate, and *n*-butanol were respectively used to extract crude ethanol extract of *Kadsura* *longipedunculata* in this experiment, and the α-amylase inhibitory activities of three fractions were tested. As a result, the ethyl acetate extract of *Kadsura* *longipedunculata* showed the best α-amylase inhibition with IC_50_ value of 59.7 μg/mL, which indicated that this fraction contained compounds with α-amylase inhibition activities. However, the α-amylase inhibitory activities of *n*-butanol and petroleum ether fractions were greater than 1000.0 μg/mL. Therefore, the ethyl acetate extract of *Kadsura* *longipedunculata* was chosen for α-amylase inhibitor screening assays. Compared with the chromatogram of ethyl acetate extract of *Kadsura* *longipedunculata* shown in Figure 1a, two peaks appeared in the chromatogram of ultrafiltrated solvent incubated with active α-amylase (Figure 1b). While, there was no peak observed in the chromatogram of ultrafiltrated solvent incubated with deactivated α-amylase at the same retention time (Figure 1c). Thus, two potential α-amylase inhibitors were considered as screened active compounds by CU.

### 2.2. Optimization of the HSCCC Solvent System

The unique separation ability of HSCCC separation for many kinds of compounds was owing to the variable two phase solvent system applied. Therefore, a suitable solvent system is vital for the separation of target compounds in HSCCC separation. In order to get a suitable solvent system for the separation of two target compounds, *K* values of two target compounds were considered as the most important parameter, which should be in the range from 0.5 to 2.0 [28]. The separation factor between the two compounds (α = *K*_1_/*K*_2_, *K*_1_ > *K*_2_) should be greater than 1.5. Moreover, short settling time (shorter than 30 s), proper retention of the stationary phase (higher than 40%), and suitable volume ratio of two phases (close to 1:1) were also necessary for the selection of solvent system [29].

The *n*-hexane–ethyl acetate–methanol–water solvent system was testified useful in separation of components in ethyl acetate extract because it could provide a broad range of polarities by modifying the volume ratio of each solvent [30]. Through a series of experiments and calculations, the *K* values of two target compounds were shown in Table 1. The *K* values of compound **2** in *n*-hexane–ethyl acetate–methanol–water (2:5:2:5 and 3:5:3:5, *v/v*) solvent systems were lower than 0.5, which could cause poor peak resolution and separation ability. While high *K* value of compound **1** in *n*-hexane–ethyl acetate–methanol–water (1:5:1:5, *v/v*) could result in long retention time and the waste of time and solvents. The separation factor (α value) was also calculated. As shown in Table 1, the α values in these solvent systems were all greater than 1.5. Accordingly, *n*-hexane–ethyl acetate–methanol–water (1.5:5:1.5:5, *v/v*) was chosen as the solvent system for HSCCC experiments.

### 2.3. HSCCC Target Guided Separation

**Figure 2:**
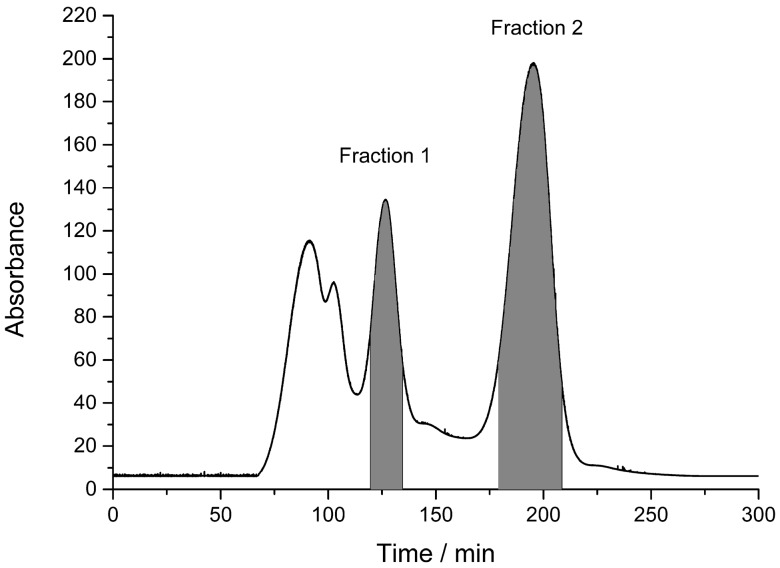
HSCCC chromatogram of *Kadsura* *longipedunculata*.

**Figure 3:**
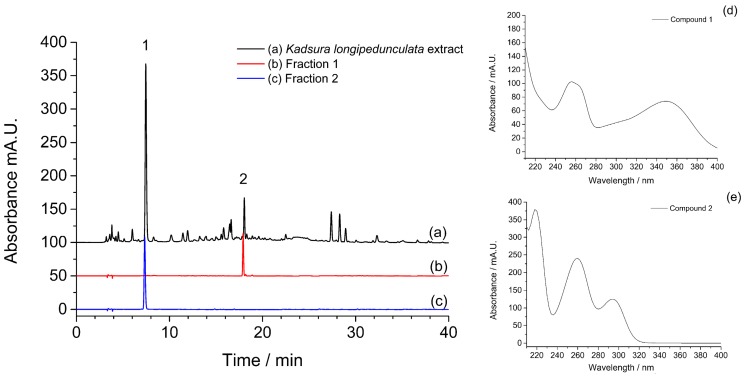
The chromatograms of (**a**) *Kadsura* *longipedunculata*; (**b**) fraction 1; and (**c**) fraction 2; and the UV spectra of (**d**) compound **1**; and (**e**) compound **2**.

The HSCCC separation was performed with the *n*-hexane–ethyl acetate–methanol–water (1.5:5:1.5:5, *v/v*) solvent system. The upper organic phase was used as stationary phase in this HSCCC separation. As is known to all, both higher rotary speed and lower flow rate could increase the retention of stationary phase and prolong the separation time during HSCCC separation [31]. In the present study, the flow rate of mobile phase, rotary speed of the separation column and sample loading were all investigated in *n*-hexane–ethyl acetate–methanol–water (1.5:5:1.5:5, *v/v*) solvent system. Considering the maximum retention of stationary phase (46.2%) and the acceptable experimental duration, a flow rate of 2.0 mL/min for the pump, a rotary speed of 900 rpm for the column and 5.0 mL for the sample loading were ultimately selected as the HSCCC separation conditions. Figure 2 presented the HSCCC chromatogram of ethyl acetate extract of *Kadsura* *longipedunculata*. As shown in Figure 2, Fraction 1 and Fraction 2 were marked and analyzed by HPLC. The chromatograms of two fractions were shown in Figure 3. The marked two fractions in HSCCC chromatogram were respectively combined and evaporated to dryness under vacuum at 50 °C. 10.5 mg of compound **1** and 13.7 mg of compound **2** from 200 mg of ethyl acetate extract were obtained with purities of 92.3% and 94.6% determined by HPLC.

### 2.4. Identification of Target Compounds

**Figure 4:**
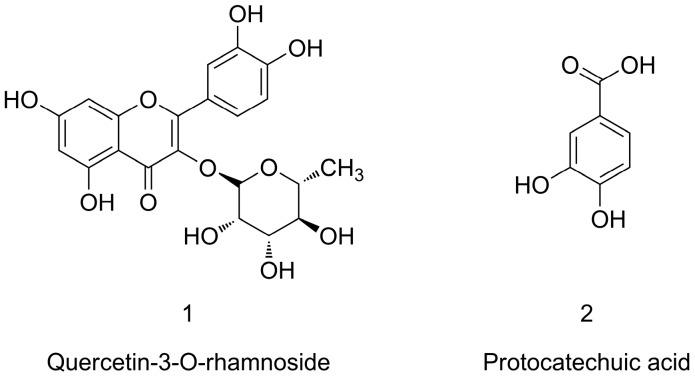
Chemical structures of two investigated compounds.

The structures of two target compounds were identified by MS, UV, and ^1^H-NMR data. The UV spectrum of compound **1** showed absorbance at 256 and 348 nm. The MS spectrum of compound **1** showed the deprotonated molecular ion [M − H]^−^ at 447 *m/z* and the characteristic ion at 301 *m/z* in the negative mode, which corresponded to the loss of a rhamnose moiety. Therefore, compound **1** was identified as quercetin-3-*O*-rhamnoside (**1**) [32]. While, the UV spectrum and the MS spectrum of compound **2** showed the UV absorbance at 260 and 294 nm, and the deprotonated molecular ion [M − H]^−^ at 153 *m/z*. According to the previous reports, the data were in agreement with literature values. The compound **2** was identified as protocatechuic acid (**2**) [33]. Their chemical structures were shown in Figure 4. Data are shown as below:

Compound **1**: UV max (nm): 256, 348; ESI-MS *m/z*: 447 [M − H]^−^, 301 [M − H − rhamnose]^−^; ^1^H-NMR (400 MHz, DMSO-*d*_6_) δ (ppm): 12.66 (s, 1H), 10.88 (s, 1H), 9.72 (s, 1H), 9.35 (s, 1H), 7.30 (d, *J* = 2.16 Hz, 1H), 7.25 (m, 1H), 6.86 (d, *J* = 8.32 Hz, 1H), 6.39 (d, *J* = 2.08 Hz, 1H), 6.20 (d, *J* = 2.08 Hz, 1H), 5.25 (d, *J* = 1.32 Hz, 1H), 4.95 (d, *J* = 4.24 Hz, 1H), 4.74 (d, *J* = 4.56 Hz, 1H), 4.63 (d, *J* = 5.72 Hz, 1H), 3.97 (s, 1H), 3.50 (m, 3H), 3.18 (m, 3H), 0.81 (d, *J* = 5.96 Hz, 3H) [34].

Compound **2**: UV max (nm): 260, 294; ESI-MS *m/z*: 153 [M − H]^−^; ^1^H-NMR (400 MHz, DMSO-*d*_6_) δ (ppm): 12.30 (s, 1H), 9.64 (s, 1H), 9.33 (s, 1H), 7.33 (d, *J* = 1.96 Hz, 1H), 7.29 (dd, *J* = 8.24, 2.00 Hz, 1H), 6.78 (d, *J* = 8.24 Hz, 1H) [35].

### 2.5. Inhibition Activity on α-Amylase of Target Compounds

In order to confirm the effectiveness of proposed method, α-amylase inhibitory activities of two isolated compounds were evaluated. As a result, quercetin-3-*O*-rhamnoside (**1**) and protocatechuic acid (**2**) exhibited inhibition on α-amylase, and the IC_50_ values of these two compounds were 28.8 and 12.5 μmol/L, respectively. These results illustrated that quercetin-3-*O*-rhamnoside (**1**) and protocatechuic acid (**2**) actually possess satisfied inhibition on α-amylase. According to current reported research and literature, the inhibition activities of protocatechuic acid on both α-glucosidase and α-amylase were reported [36,37]. It proved that screening using CU-LC-MS for α-amylase inhibitors from natural products was efficient and effective.

## 3. Materials and Methods

### 3.1. Materials

*Kadsura longipedunculata* was bought from Hunan Tianjian Chinese Medicine Pieces Co., Ltd. (Changsha, China) and identified by Prof. Aiping Xiao. A voucher specimen (N160108) has been deposited in the Institute of Bast Fiber Crops, Chinese Academy of Agricultural Sciences (Changsha, China). α-Amylase (≥10 units/mg, from porcine pancreas), 3,5-Dinitrosalicylic acid (DNS), and soluble starch were acquired from Sigma-Aldrich (Saint Louis, MO, USA). Acetonitrile in HPLC grade was purchased from Tedia Inc. (Phoenix, AZ, USA). Pure water was obtained from a Milli-Q water purification system (Millipore, Billerica, MA, USA). All of the other chemicals were of analytical grade and obtained from Sinopharm Chemical Reagent Co., Ltd. (Shanghai, China).

### 3.2. Preparation of Kadsura longipedunculata Extracts

Dried *Kadsura* *longipedunculata* (50.0 g) was soaked and extracted thrice with 300 mL of ethanol solution (90% *v/v*) at 90 °C to yield the crude extract. Then, the ethanol extracts were evaporated to remove the solvent under reduced pressure. One hundred milliliters of water was added into the condensed residue (4.03 g) in order to form an aqueous solution. The aqueous solution was successively extracted with the same volume of petroleum ether, ethyl acetate, and *n*-butanol according to the polar order. After that, the three extracts of *Kadsura* *longipedunculata* were evaporated to remove the solvents and got residues (petroleum ether extract: 0.60 g, ethyl acetate extract: 0.58 g and *n*-butanol extract: 2.03 g), respectively. The three extracts of *Kadsura* *longipedunculata* was then dissolved in 100 mL of water to form solution. Finally, the solution was filtered to ensure the clarification of sample solution by a 0.45 μm membrane and stored at 4 °C prior to use.

### 3.3. HPLC Analysis Conditions

In this study, Agilent 1260 Infinity system (Agilent Technologies Inc., Santa Clara, CA, USA) was used in the HPLC analysis. A Waters Xbridge™ C18 reverse phase column (250 mm × 4.6 mm i.d., 5 μm) was used as the column performing HPLC separation (Waters, Milford, MA, USA). Many experimental conditions such as mobile phase compositions, flow rates, column temperature, gradient elution methods and detection wavelength were tested. As a result, the column temperature was maintained at 25 °C. The flow rate was controlled at 0.8 mL/min. The mobile phase consisted of solvent A (water containing 0.4% *v/v* acetic acid) and solvent B (acetonitrile containing 0.4% *v/v* acetic acid) with gradient elution mode: 0–5 min, 90% A; 5–30 min, 90%–40% A; 30–40 min, 40%–10% A. The scan wavelength of diode array detector was set from 200–400 nm, and the detection wavelength of chromatogram was set at 254 nm.

### 3.4. Screening of α-Amylase Inhibitors from Kadsura longipedunculata

The α-amylase inhibition assay was conducted by CU. The ethyl acetate extract of *Kadsura longipedunculata* (100 μL, 5.0 mg/mL) was incubated with 100 μL of α-amylase solution (40.0 μg/mL) at room temperature. After 90 min incubation with enzyme solution, the mixture containing samples and enzymes was poured into a Nanosep MF centrifugal filter and CU was performed in a centrifuge (Allegra 64R, Beckman Coulter, Brea, CA, USA). The process was conducted at 4 °C and 10,000 rpm for 10 min. Then 200 μL of water was added to remove the unbound constituents. Finally, the bound α-amylase inhibitors were released by adding 200 μL of methanol and centrifuging at 4 °C and 10,000 rpm for 10 min. The eluent was collected and stored at 4 °C prior to use without any treatment, while using deactivated α-amylase solution as control.

### 3.5. HSCCC Target Guided Separation

The procedures for the determination of the *K* value were as follows: some sample was added to the two tested phases of the solvent system and thoroughly shaken until the phase equilibrium reached. After that, the upper phase solution and lower phase solution were taken out and evaporated to dryness, respectively. Subsequently, the residues of upper phase and lower phase were redissolved in 2.0 mL of methanol solution and filtrated for HPLC analysis. The *K* value of target compound was calculated as follows:

*K* = S_1_/S_2_

where S_1_ is the peak area of a certain compound in upper phase and S_2_ is the peak area of a certain compound in lower phase.

In this study, the solvent system used for HSCCC target guided separation was comprised by *n*-hexane–ethyl acetate–methanol–water at a volume ratio of (1.5:5:1.5:5, *v/v*). Each component of the proposed solvent system was added to a separatory funnel and thoroughly equilibrated by shaking. The two phases of solvents were separated and stored. The solvents should be degassed ultrasonically for more than 30 min before usage in order to prevent the formation of bubbles and the interference to detector during the HSCCC separation. For the HSCCC target guided separation, 200 mg of the ethyl acetate extract of *Kadsura longipedunculata* residues were dissolved in the mixed solution containing the same amount of upper phase and lower phase (5.0 mL, 1:1, *v/v*).

A TBE-300A HSCCC (Tauto Biotechnique Company, Shanghai, China) was used for HSCCC separation in this study. The apparatus consisted of three polytetrafluoroethylene multilayer coil columns connected in series (inner diameter of the tubing: 1.6 mm, total column volume: 260 mL) and a 20 mL sample loop. The revolution radius or the distance between the holder axis and the central axis of the centrifuge (R) was 5 cm. The β values of the multilayer coil ranged from 0.5 at the internal terminal to 0.8 at the external terminal (β = r/R, where r is the distance from the coil to the holder shaft). The revolution speed could be regulated in the range between 0 and 1000 rpm by a speed controller. At first, the upper phase was pumped into the machine in order to fill the multilayer coil column. After that, the column was rotated at 900 rpm. The lower phase was then pumped into the column with a flow rate of 2.0 mL/min. When the system reached equilibrium, 5.0 mL of sample solution was injected via the injection valve. During the HSCCC separation, the temperature of the system was maintained at 25 °C. The effluent was collected manually every 5 min for each tube under the constantly detection at 254 nm with a UV detector. After 300 min of separation, about 300 mL of upper phase solvents and 800 mL of lower phase solvents were used. At the end of separation, the column was stopped and nitrogen gas was used to blow out the solution remaining in the column. The collected fractions were numbered and analyzed by HPLC. The purity of the compound was analyzed by HPLC, combined with the peak area normalization method, which calculated the area of each peak in the chromatogram as a percentage of the total area of all peaks and provided an approximate value of the relative purity.

### 3.6. Identification of α-Amylase Inhibitors

The screened potent α-amylase inhibitors from HSCCC separation of *Kadsura* *longipedunculata* extract were analyzed by HPLC-MS. HPLC analysis was performed on an Agilent 1290 Infinity LC system (Agilent Technologies Inc.) under the same HPLC condition. Triple quadrupole tandem mass analysis was accomplished using an Agilent 6460 Triple Quadrupole LC-MS (Agilent Technologies Inc.). In this experiment, an electrospray ionization (ESI) interface was equipped and worked in negative ionization mode. Full scan mode was set as the mass detection mode from 100 *m/z* to 1000 *m/z*. ^1^H-NMR experiments were conducted with an AVANCE III 400 M spectrometer (Bruker Corporation, Karlsruhe, Germany) operating at 400 MHz, while deuterated DMSO was used as the solvent, and chemical shifts (δ, ppm) were reported with reference to tetramethylsilane.

## 4. Conclusions

An analysis method utilizing HSCCC target guided by CU-LC-MS was established and it was applied in screening and isolating α-amylase inhibitors in *Kadsura* *longipedunculata* extract. Two compounds were considered as the potential inhibitors in the CU-LS-MS analysis, and then were separated during one time HSCCC separation with high purities. According to the UV and MS data, the structures of two separated compounds were compared with the reported compounds and finally confirmed as quercetin-3-*O*-rhamnoside and protocatechuic acid. The HSCCC separation target guided by CU-LC-MS realized rapid and effective isolation of potential active compounds after the screening and identification. Results demonstrated that the target guided HSCCC with CU-LC-MS was a good method and was suitable for discovering bioactive and active compounds from natural products.

## Figures and Tables

**Table 1:** *K* values of two target compounds in different solvent systems.

Solvent System	Ratio (*v*/*v*)	*K* Value		α
		Compound 1	Compound 2	
*n*-Hexane–Ethyl acetate–Methanol–Water	1:5:1:5	2.537	1.507	1.683
	1.5:5:1.5:5	1.488	0.689	2.160
	2:5:2:5	0.912	0.358	2.547
	3:5:3:5	0.329	0.072	4.569
